# The mediating role of health literacy between the presence of chronic disease and psychological distress among older persons in Xi’an city of China

**DOI:** 10.1186/s12889-023-17315-x

**Published:** 2023-12-18

**Authors:** Kun Guo, Jing Ouyang, Halimatus Sakdiah Minhat

**Affiliations:** 1 College of Humanities and management, Shaanxi university of Chinese medicine, Xianyang, Shaanxi province China; 2 Department of Community Health, Faculty of medicine and health sciences, University Putra Malaysia, Kuala Lumpur, Malaysia; 3 Malaysian Research Institute on Ageing, University Putra Malaysia, Serving, Selangor, 43400 Malaysia

**Keywords:** Chronic Disease, Cognitive, Negative mental health, Older adults, China

## Abstract

**Background:**

The increased number of older persons in China, and the prevalence of most chronic diseases raised with age significantly increased the total disease burden. When a person ages, psychological distress happens when they are faced with stressors that they cannot cope with. Psychological distress refers to non-specific symptoms of depression, anxiety, and stress. Health literacy influences several health outcomes, such as emotional functioning among the population. The primary purpose of this study is to examine the mediator role of health literacy between the presence of chronic disease and psychological distress among older persons living in Xi’an city. Thus, this study used the Cognitive Behavior Theory (CBT) as a combination of the basic behavioral and cognitive psychology principles to explain the cognitive processes associated with psychological distress.

**Methods:**

This study employs a quantitative research design using a cross-sectional survey of 300 older persons over 60 years living in the six urban districts of Xi’an city. Data were collected using the Health Literacy Questionnaire (HLQ) and the Depression Anxiety Stress Scale (DASS-21). This study employed descriptive statistics and inferential methods to analyze the data. The inferential methods applied structural equation modeling (SEM) to test the hypothesis of the mediator role of health literacy between the presence of chronic disease and psychological distress.

**Results:**

In this study, chronic disease had an effect on health literacy among older persons living in Xi’an city (β=-0.047, *p* < 0.01); chronic disease impact on psychological distress among older persons living in Xi’an city (β = 0.047, *p* < 0.01); health literacy was identified effect on psychological distress among older persons in Xi’an city (β=-0.738, *p* < 0.001); health literacy as a partial mediator between chronic disease and psychological distress (β = 0.07, *p* < 0.01).

**Conclusion:**

Psychological distress among older persons is affected by chronic disease and health literacy. Health literacy had a partial mediating effect on the presence of chronic disease and psychological distress. Improved health literacy measures should be considered when treating older persons with psychological distress.

## Introduction

In the past few decades, more and more people have been living longer, and the population of older persons has increased. Most people today live beyond 60 for the first time in history. China’s population is aging faster than almost any other country in modern history [1]. In 2017, the percentage of Chinese residents aged 60 was 17.3%, or more than241 million [2]. The population of people over 60 years old in China is projected to reach 28% by 2040 [3]. The rapid increase in the proportion of older persons in China can be attributed to the one-child policy and longer life expectancy [4, 5]. With the acceleration of urbanization in China and the increase of the older population living in urban areas, the government needs to increase investment in medical and old-age care. To ensure society’s development, city residents over 60 need to be given more attention.

The prevalence of most chronic diseases rises with age. In 2013, nearly 50% (100 million) of older people in China experienced chronic diseases [6]. In China, the prevalence rate of hypertension among persons aged 60 and above was 66.9%; the prevalence of diabetes among persons aged 60 and up was 19.6%; the prevalence of myocardial infarction among persons aged above 60 years was 1.3%; the prevalence of arthritis was 25% in the person aged 60 years and above; the prevalence of Chronic Obstructive Pulmonary Disease (COPD) was 15.5% in the person aged 60 years and above; the prevalence of asthma was 3.4% among persons aged 60 years and above [3]. The increased number of older persons in the population, as forecast, significantly increased the total disease burden.

One problem associated with older persons is psychological distress. Depression is a psychological distress affecting elders worldwide and is known to be a comorbidity of many chronic illnesses and a significant predictor of mortality among older adults [7]. The prevalence of depression among older persons in China has ranged from 14.8 to 23.6%, according to a systematic review and meta-analysis [8]. One of the psychological distresses among the world’s population is anxiety, which increases with the aging of the population [9]. A comprehensive review estimated the prevalence of anxiety in older persons ranged from 3.2 to 14.2% [10]. Compared with other age groups, stress indicates more severe and intense health effects for older adults, and perceived stress was the strongest predictor of anxiety [11]. A study that used data from the China Health and Nutrition Survey in 2015 found that the prevalence of stress was 22.13% among older adults [12]. Thus, older persons often have psychological distress, such as depression, anxiety, and stress during this period of life.

One commonly used scale for detecting psychological distress is the Depression Anxiety and Stress Scale (DASS) [13]. Since the DASS-21 introduction in 1995, it has been widely used to assess depression, anxiety, and stress among adults and has shown the DASS-21 has strong validity [13]. The Chinese version of DASS-21 is a common instrument for screening psychological distress during the past week [14]. There is an element of hopelessness, self-deprecation, low positive affect, and a devaluation of life in the depression subscale; the anxiety subscale is related to physiological hyperstimulation and a subjective consciousness of anxious affect, while the stress subscale is comprised of relaxation difficulties, tension, impatience, irritability, and restlessness. These characteristics are common among the subscales of DASS-21, including negative affect, emotional distress, and changes in hypothalamus-pituitary-adrenal physiology [15]. There are scholars who developed the psychometric structure of DASS in statistical terms, proposing that depression, anxiety, and stress could be sub-dimensions of a higher-order mental factor called psychological distress [16]. The unique advantage of using DASS with a psychometric construct of psychological distress is that simultaneous interactions/complications of depression, anxiety, and stress can be examined in data analysis. This allows researchers to generate more comprehensive findings in their study. Thus, this study uses the Chinese version of DASS-21 to examine psychological distress among older persons in Xi’an city.

WHO defines health literacy as “the cognitive and social skills that determine the motivation and ability of individuals to gain access to, understand, and use information in ways that promote and maintain healthy mental health” [17]. Health literacy influences several health outcomes, such as physical functioning, emotional functioning, healthcare utilization, key decision-making outcomes, and self-care management among the population. Health literacy includes knowledge of the preventive measures, symptoms, treatment modes, and treatment locations of psychological disorders. It also includes taking action to support individuals or others experiencing mental health conditions [18]. Health literacy may impact older persons more than other age groups [19]. Limited health literacy would increase the risk of poor physical functioning, limitations of daily activities, poor mental health status, and not achieving optimal health outcomes among older persons [20]. According to a previous study, older persons with low health literacy levels will incur more medical fees in daily life. They will have more emergency visits, hospital admissions, and less access to health care. Thus, the Shanghai Declaration of 2016 recognizes health literacy as one of the critical health promotion pillars for achieving sustainable development goals [21]. To achieve the target, community and government organizations must integrate health literacy as a cornerstone of all national health agendas [22]. The main goal of health literacy is to raise individuals’ awareness of health/disease status to achieve healthy outcomes. There was a range of health literacy measurement instruments. However, most tools do not reflect the multidimensional definition of health literacy [23]. The Health Literacy Questionnaire (HLQ) was developed in Australia in 2012, and it covers nine conceptually distinct areas of health literacy [24]. The HLQ was developed to address the shortcomings of previous tools [25]. There was a study that proved the Chinese version of the HLQ has strong construct and content validity and high composite reliability when applied to older adults in Changsha City, China [26]. Therefore, this study uses the HLQ to evaluate health literacy among older persons in Xi’an city.

Several studies have attempted to prove that health literacy affects health outcomes [27, 28]. In a previous study, health literacy was linked to depression but did not remain an independent risk factor for depression, unlike personal characteristics associated with depression symptoms [29]. Although it is well established that limited health literacy impacts mental health conditions, the mechanisms by which this occurs remain unclear. One potential mechanism is identifying the factors associated with health literacy and psychological distress among older persons.

This study was conducted in Xi’an city, Shaanxi Province, China. Shaanxi province has the 16th highest population in China. Xi’an is a major city in north-western China with a population of approximately 7.64 million [30]. Xi’an city is the capital of Shaanxi province, one of the most ancient cities in China, with more than 3100 years of history [31]. Xi’an is the largest city in northwest China, with an area of 10,108 km^2^, and this city has 13 districts. Xi’an covers six central urban districts, which are Xin Cheng, Bei Lin, Lian Hu, Ba Qiao, Wei Yang, and Yan Ta; five suburban districts, which are Yan Liang, Lin Tong, Chang An, Gao Ling, and Hu Yi; and two rural countries which are Lan Tian, and Zhou Zhi [32]. With the development of urbanization, older persons become a vulnerable group in urban areas. A study recruited 360 older persons in Xi’an in 2016 and found that social support plays a decisive role in older persons’ mental health conditions [33]. The relationship between health literacy and mental health outcomes among older persons in Xi’an city has yet to be studied. Determining the relationship between health literacy and psychological distress could aid the development of more targeted interventions because it can improve the quality of life among older persons.

**Fig. 1:**
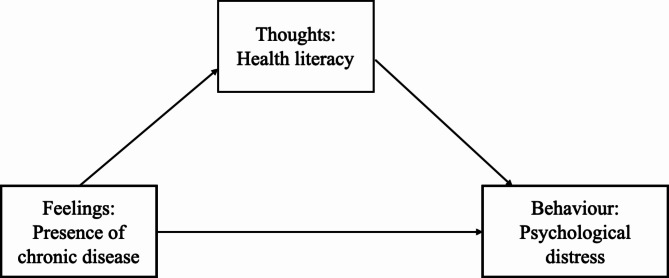
The theoretical model of the mediating role of health literacy between the presence of chronic disease and psychological distress among older persons in Xi’an city, according to CBT

To better understand the phenomenon of the present study, a relevant theory must be established. Similar to a previous study investigating the predictors of self-care behavior among homebound older persons using the Health Empowerment theory as a framework [34]. In this regard, Cognitive Behavior Theory (CBT) was used as a theoretical framework to explain the characteristics of respondents according to their feelings, thoughts, and behavior constructs. People diagnosed with a chronic disease may feel pain physically or feel confused and emotional. Therefore, in this study, the presence of chronic disease constitutes personal feelings in CBT. Health literacy is the cognitive ability to understand and use information to maintain health, and health literacy constitutes the thoughts in CBT. Psychological distress refers to unsatisfactory past and present relationships that result in maladaptive behavior. Psychological distress suggests deficits in social skills, including less adaptive nonverbal behavior [35]. Therefore, in this study, psychological distress constitutes the behavior in CBT. The theory of CBT was chosen because it sufficiently considers the variables in the study to meet its objective. CBT is based on the combination of basic principles from behavioral and cognitive psychology. In CBT models, cognitive processes, in the form of meanings, judgments, appraisals, and assumptions associated with specific life events, are the primary determinants of one’s feelings and actions in response to life events and thus either facilitate or hinder the process of adaptation. Dobson and Dozois (2001) recognize that CBT is characterized by the following assumptions: “(a) Cognitive activity affects behavior, (b) cognitive activity may be monitored and altered, (c) behavior change may be achieved through cognitive change [36]. CBT focuses on the rationality of one’s thinking patterns and the connections between thoughts, feelings, and behaviors. According to CBT, it is grounded in the belief that how a person perceives events determines how they will act [37]. CBT believes people can adjust their thoughts, and their thoughts will directly influence their emotions and behavior. Thus, CBT is available to explain health literacy as the mediating effect on the presence of chronic diseases and psychological distress among older persons (as shown in Fig. 1).

The objective of this study: To determine the mediating role of health literacy between the presence of chronic disease and psychological distress among older persons in Xi’an City through the application of CBT.

The hypothesis of this study:

### #1

There is a significant relationship between the presence of chronic disease and health literacy among older persons in Xi’an City.

### #2

There is a significant relationship between the presence of chronic disease and psychological distress among older persons in Xi’an City.

### #3

There is a significant relationship between health literacy and psychological distress among older persons in Xi’an City.

### #4

Health literacy is a significant mediator role between the presence of chronic disease and psychological distress among older persons in Xi’an City.

## Methodology

### Population

This study utilized a cross-sectional study design. China is the largest developing country in the world and uses the cut-off point of 60 years old to define an individual as an older person based on the definition proposed by the United Nations [38]. Xi’an city consists of six urban districts: Xin Cheng, Bei Lin, Lian Hu, Ba Qiao, Wei Yang, and Yan Ta [32]. This study employed structural equation modeling (SEM) analysis as the main analysis component. According to Hair et al. (2010), the estimation of the minimum sample size for SEM depends on the number of constructs included in the model. A model containing five or fewer constructs requires a minimum sample size of 100; a model containing seven constructs or less requires a minimum sample size of 150; a model containing seven or more constructs requires a minimum sample size of 300 [39]. Based on these pieces of scientific evidence, a minimum sample size of 300 was needed to run the path analysis. Therefore, this study utilized 300 as the final sample size.

This study recruited 300 older persons who can understand and communicate in Chinese Mandarin and older persons above 60 who live in the six urban districts of Xian city. This study excluded non-hearing-wearing -aid wearing hearing-impaired older persons because these people who are hearing impaired have either a partial or a total inability to hear sound and cannot communicate with them smoothly.

### Ethical considerations

This study received ethical approval from the Ethic Committee for Research Involving Human Subjects University Putra Malaysia or Jawatankuasa Etika Universiti untuk Penyelidikan Medlibatkan Manusia (JKEUPM). This study refers to the PROJECT REF. NO: JKEUPM-2020-328. All eligible participants were informed about the study. A written information sheet and a consent form were provided for participants who wished to participate. Participants were clearly informed that their participation was voluntary and that refusing to participate would not result in adverse consequences. All personal information was kept anonymous throughout the process, and all questionnaire data was stored in a password-protected cabinet.

### Procedures

The survey was conducted between 1 January and 25 March 2021. Initially, this study used stratified proportionate size sampling in order to conduct probability sampling by randomly recruiting older persons from each central urban district using the calculated proportion (refer to Table 1). However, due to the large number of older persons in each central district and the COVID-19 pandemic during data collection. Data were collected by interviewing older persons face-to-face using a convenient sampling method based on the calculated proportion (as shown in Table 1).

**Table 1:** Summary of the older persons proportional sample size in each central urban district

No.	District	Proportion	Sample size
1	Xin Cheng	128,618/810,990 = 15.86%	48
2	Bei Lin	152,806/810,990 = 18.84%	56
3	Lian Hu	182,317/810,990 = 22.48%	68
4	Yan Ta	162,635/810,990 = 20.05%	60
5	Wei Yang	94,095/810,990 = 11.60%	35
6	Ba Qiao	90,519/810,990 = 11.16%	33
Total		100%	300

Source: Data of each district population in this table are obtained from the seventh China population census (2020)

### Measurements

Questionnaires can be used as the sole research instrument (such as in a cross-sectional survey) or in epidemiological studies [40]. In this study, participants were interviewed face-to-face to complete the questionnaire. There were three sections to the questionnaire: A, B, and C. Section A asked about the respondents’ information, such as age, gender, income, marital status, and presence of chronic disease. The definitions of personal factors were as follows: gender (male = 1, female = 2), marital status (Married = 1, Unmarried/divorced/ widowed = 2), income (0 ~ 2999 RMB; 3000 ~ 5999 RMB; 6000 ~ RMB). The respondents were initially asked if they suffered from any chronic disease, and then they had to identify the type of the chronic disease. Chronic disease in this study refers to the presence of any disease or condition that has lasted for three months or longer and may worsen over time [41].

Section B is the health literacy questionnaire (HLQ), divided into nine scales with44 items. The nine scales are (1) Feeling understood and supported by healthcare providers (four items); (2) Having sufficient information to manage my health (four items); (3) Actively managing my health (five items); (4) Social support for health (five items); (5) Appraisal of health information (five items); (6) Ability to engage with healthcare providers actively (five items); (7) Navigating the healthcare system (six items); (8) Ability to find good health information (five items); (9) Understand health information well enough to know what to do (five items) [42]. Items for the first to fifth of the nine scales are rated on a 4-point Likert scale, ranging from ‘strongly disagree’ to ‘strongly agree’, and items for scales sixth to ninth use a 5-point Likert scale, ranging from ‘cannot do’ to ‘very easy’. This study used the Chinese version of the HLQ, which was provided by the developers of the instrument. The Chinese version of HLQ used in this study has been validated and is highly reliable. The development and validation study showed that the HLQ has strong structural validity, reliability, and acceptability [43]. In this study, the HLQ had adequate reliability with Cronbach alphas of 0.983 for the overall scale. The Cronbach’s alpha measurement is 0.805 for feeling understood and supported by healthcare providers; 0.813 for having sufficient information to manage my health; 0.78 for actively managing my health; 0.823 for the social health support; 0.881 for the appraisal of health information; 0.828 for engaging effectively with healthcare providers; 0.88 for the ability to navigate the healthcare system; 0.808 for finding reliable health information; 0.799 for the understand health information well enough to know what to do.

Section C is the Depression Anxiety Stress Scale (DASS-21), which includes three scales and has 21 items [44]. According to Lovibond, S. H. & Lovibond, P. F. (1995), the manual of DASS-21 outlines an individual’s depression, anxiety, and stress level based on each subscale’s score [45]. The rating choices are ‘never applied to oneself’ (0 points), ‘some degree/some of the time’ (1 point), ‘considerable degree/a good part of the time’ (2 points), and ‘very much/most of the time’(3 points). Higher scores indicate more psychological distress [46]. The Chinese version of DASS-21 was used in this study, and the Chinese version of DASS-21 was translated by Yi et al. [47]. The Chinese version of DASS-21 has been validated and is highly reliable. In this study, the DASS-21 had adequate reliability with Cronbach alphas of 0.952 for the overall scale. The Cronbach’s alpha of 0.886 for depression, 0.891 for anxiety, and 0.884 for stress. Therefore, the DASS-21 instrument used in this study has high validity and reliability.

### Data analysis

In this study, descriptive and inferential analysis were used to analyze data. The data was analyzed using the IBM SPSS Statistics version 26 and R software. The descriptive statistical analysis describes the personal factors and the prevalence of depression, anxiety, and stress among respondents. Inferential statistics were employed to infer the population from which samples were drawn for this study. Structural equation modeling (SEM) was applied to test the hypothesized model. SEM is a multivariate technique that combines Confirmatory factor analysis (CFA), correlation, multiple linear regression, and path analysis. The SEM analysis was done by lavaan, which is an R package for SEM. Lavaan package of R is an acronym for latent variable analysis, and its name reveals the long-term goal: to provide a collection of tools that can be used to explore, estimate, and understand a wide family of latent variable models, including factor analysis, structural equation, longitudinal, multilevel, latent class, item response, and missing data models [48]. SEM technique was used in this study because it is a more robust test of mediation relationships than multiple regression analysis [49]. Three levels of analysis were conducted in SEM in this study, and the first level was CFA, followed by the measurement model and then a structural model. A *P*-value < 0.05 was defined as statistically significant. Model fit was assessed through several commonly reported fit statistics, such as RMSEA (root mean square error of approximation) ≤ 0.08, GFI (Goodness of fit index) ≥ 0.90, CFI (comparative fit index) ≥ 0.90, NFI (normed fit index) ≥ 0.90, TLI (tucker-lewis index) ≥ 0.90, SRMR (standardized root mean square residual) ≤ 0.08, Chi-square (χ²) ≥ 0.5, Chi-sq/df ≤ 5.0 [39].

## Results

### Characteristics of the participants

The descriptive information of 300 participants was provided in Table 2. All the respondents were above 60 years old. The mean age of respondents was 68.94 years (SD = 7.657), and approximately half were female (51.7%). Most participants were married (63%), a majority of the respondents earned 3000 ~ 5999 RMB a month (41.7%), and the majority of the respondents had chronic disease (53.3%). The average scores and standard deviations of HLQ scores are presented in Table 3. For the first five scales, the “Social support for health” scale had the highest average score (Mean = 2.60, SD = 0.45). For the remaining scales, “Ability to actively engage with healthcare professionals actively” had the highest average score (Mean = 2.90, SD = 0.56). Table 3 is not intended to compare the scores of the nine HLQ scales. Instead, it is to give the reader an overall idea about the respondents’ health literacy scores.

**Table 2:** Participants characteristics (N = 300)

Variable		Frequency	Percentage (%)
Age	60 ~ 69	201	67
	70 ~ 79	60	20
	≥ 80	39	13
Gender	Male	145	48.3
	Female	155	51.7
Marital status	Married	189	63
	Unmarried/divorced/widowed	111	37
Income monthly	0 ~ 2999 (RMB)	107	35.7
	3000 ~ 5999 (RMB)	125	41.7
	6000~ (RMB)	68	22.7
Presence of chronic disease	Not have chronic disease	140	46.7
	Have chronic disease	160	53.3

**Table 3:** HLQ scores for respondents (N = 300)

HLQ score	Mean	SD
Section one: Scales of agreement. Range 1 (lowest)to 4(highest)		
HL1.Feeling understood and supported by healthcare professionals	2.41	0.44
HL2.Having sufficient information to manage my health.	2.42	0.45
HL3.Actively managing my health	2.52	0.42
HL4.Social support for health	2.60	0.45
HL5.Appraisal of health information	2.55	0.46
Section two: Scales of capabilities. Range 1 (lowest)to 5(highest)		
HL6.Ability to actively engage with healthcare professionals.	2.90	0.56
HL7.Navigating the healthcare system.	2.85	0.53
HL8.Ability to find good health information.	2.85	0.55
HL9.Understand health information enough to know what to do.	2.87	0.55

### Confirmatory factor analyses

CFA is the first step of SEM analysis and considers data preparation before actual SEM analysis [50]. The model fit test can determine how well the model fits the sample data. Two criteria are used to examine model fit, including individual factor loadings and fit indices [51]. The value of standardized factor loading must be greater than 0.6, and the indicators lower than 0.6 must be deleted. Table 4 shows the measurement properties of reflective latent constructs. An alternative f internal consistency reliability measure is recommended to avoid the emergence of bias called composite reliability (CR). Composite reliability must be above 0.6. CR below 0.6 may compromise the validity of the questionnaire. Therefore, the statement that increases the probability of error must be removed. The internal consistency reliability is also supported by the fact that Cronbach’s alpha for reflective constructs in the study is more significant than 0.6 [52]. Based on the current study’s findings, the CR stands between 0.750 and 0.892, as shown in Table 5.

**Table 4:** Correlation of latent variables and discriminant validity (Fornell-Larcker)

	HL1	HL2	HL3	HL4	HL5	HL6	HL7	HL8	HL9	Depression	Anxiety	Stress
HL1	**0.715**											
HL2	0.464	**0.722**										
HL3	0.480	0.480	**0.708**									
HL4	0.536	0.537	0.556	**0.696**								
HL5	0.271	0.271	0.280	0.313	**0.773**							
HL6	0.230	0.230	0.238	0.266	0.521	**0.705**						
HL7	0.259	0.259	0.268	0.300	0.587	0.499	**0.722**					
HL8	0.237	0.238	0.246	0.275	0.539	0.457	0.515	**0.677**				
HL9	0.247	0.248	0.256	0.286	0.288	0.245	0.276	0.253	**0.691**			
Depression	0.192	0.192	0.199	0.222	0.224	0.190	0.214	0.196	0.458	**0.727**		
Anxiety	0.200	0.200	0.207	0.231	0.233	0.198	0.223	0.204	0.476	0.370	**0.736**	
Stress	0.234	0.234	0.243	0.271	0.273	0.232	0.261	0.240	0.559	0.434	0.451	**0.722**

**Table 5:** Measurement properties of reflective latent constructs

Construct	Item	Before modification					After modification				
		Estimate	SMC	Composite reliability	Average variance extracted	Cronbach’s alpha	Estimate	SMC	Composite reliability	Average variance extracted	Cronbach’s alpha
HL1	Item1	0.681	0.464	0.807	0.511	0.805	0.681	0.464	0.807	0.511	0.805
	Item2	0.681	0.464				0.681	0.464			
	Item3	0.705	0.497				0.705	0.497			
	Item4	0.788	0.621				0.788	0.621			
HL2	Item1	0.783	0.613	0.813	0.522	0.813	0.783	0.613	0.813	0.522	0.813
	Item2	0.665	0.442				0.665	0.442			
	Item3	0.75	0.563				0.75	0.563			
	Item4	0.687	0.472				0.687	0.472			
HL3	Item1	0.768	0.590	0.789	0.432	0.78	0.768	0.590	0.750	0.501	0.746
	Item2	0.597	0.356				-	-			
	Item3	0.62	0.384				0.62	0.384			
	Item4	0.728	0.530				0.728	0.530			
	Item5	0.549	0.301				-	-			
HL4	Item1	0.749	0.561	0.824	0.484	0.823	0.749	0.561	0.824	0.484	0.823
	Item2	0.695	0.483				0.695	0.483			
	Item3	0.687	0.472				0.687	0.472			
	Item4	0.669	0.448				0.669	0.448			
	Item5	0.676	0.457				0.676	0.457			
HL5	Item1	0.76	0.578	0.881	0.598	0.881	0.76	0.578	0.881	0.598	0.881
	Item2	0.772	0.596				0.772	0.596			
	Item3	0.747	0.558				0.747	0.558			
	Item4	0.795	0.632				0.795	0.632			
	Item5	0.79	0.624				0.79	0.624			
HL6	Item1	0.722	0.521	0.832	0.497	0.828	0.722	0.521	0.832	0.497	0.828
	Item2	0.678	0.460				0.678	0.460			
	Item3	0.652	0.425				0.652	0.425			
	Item4	0.74	0.548				0.74	0.548			
	Item5	0.73	0.533				0.73	0.533			
HL7	Item1	0.729	0.531	0.902	0.610	0.88	0.729	0.531	0.844	0.521	0.821
	Item2	0.731	0.534				0.731	0.534			
	Item3	0.754	0.569				0.754	0.569			
	Item4	0.65	0.423				0.65	0.423			
	Item5	0.741	0.549				0.741	0.549			
	Item6	1.026	1.053				-	-			
HL8	Item1	0.725	0.526	0.808	0.458	0.808	0.725	0.526	0.808	0.458	0.808
	Item2	0.703	0.494				0.703	0.494			
	Item3	0.661	0.437				0.661	0.437			
	Item4	0.634	0.402				0.634	0.402			
	Item5	0.657	0.432				0.657	0.432			
HL9	Item1	0.687	0.472	0.803	0.451	0.799	0.687	0.472	0.785	0.478	0.782
	Item2	0.684	0.468				0.684	0.468			
	Item3	0.775	0.601				0.775	0.601			
	Item4	0.611	0.373				0.611	0.373			
	Item5	0.586	0.343				-	-			
DEPRESSION	Item1	0.661	0.437	0.887	0.529	0.886	0.661	0.437	0.887	0.529	0.886
	Item2	0.714	0.510				0.714	0.510			
	Item3	0.766	0.587				0.766	0.587			
	Item4	0.705	0.497				0.705	0.497			
	Item5	0.757	0.573				0.757	0.573			
	Item6	0.732	0.536				0.732	0.536			
	Item7	0.752	0.566				0.752	0.566			
ANXIETY	Item1	0.745	0.555	0.892	0.541	0.891	0.745	0.555	0.892	0.541	0.891
	Item2	0.781	0.610				0.781	0.610			
	Item3	0.767	0.588				0.767	0.588			
	Item4	0.712	0.507				0.712	0.507			
	Item5	0.711	0.506				0.711	0.506			
	Item6	0.677	0.458				0.677	0.458			
	Item7	0.748	0.560				0.748	0.560			
STRESS	Item1	0.681	0.464	0.884	0.521	0.884	0.681	0.464	0.884	0.521	0.884
	Item2	0.713	0.508				0.713	0.508			
	Item3	0.715	0.511				0.715	0.511			
	Item4	0.78	0.608				0.78	0.608			
	Item5	0.766	0.587				0.766	0.587			
	Item6	0.67	0.449				0.67	0.449			
	Item7	0.723	0.523				0.723	0.523			

### Discriminant validity

Discriminate validity refers to the situation in which a construct is correctly differentiated from another construct by practical criteria. Discriminate validity can be determined through the examination of the model’s Fornell-Larcker and cross-loading criteria. Fornell-Larcker refers to a comparison of each construct’s AVE with squared correlations between the construct and other constructs in the model’s. Through this comparison, discriminant validity can be evaluated. That is, a construct with an AVE that exceeds the squared correlations among the constructs is considered to have adequate discriminant validity (Fornell & Larcker, 1981). As shown in Table 4, the results indicated that the values of AVE in each factor are much higher than their squared correlation. Consequently, all the constructs have adequate discriminatory validity.

### The mediating effect of health literacy on the association between the presence of chronic disease and psychological distress among older persons in Xi’an city

**Fig. 2:**
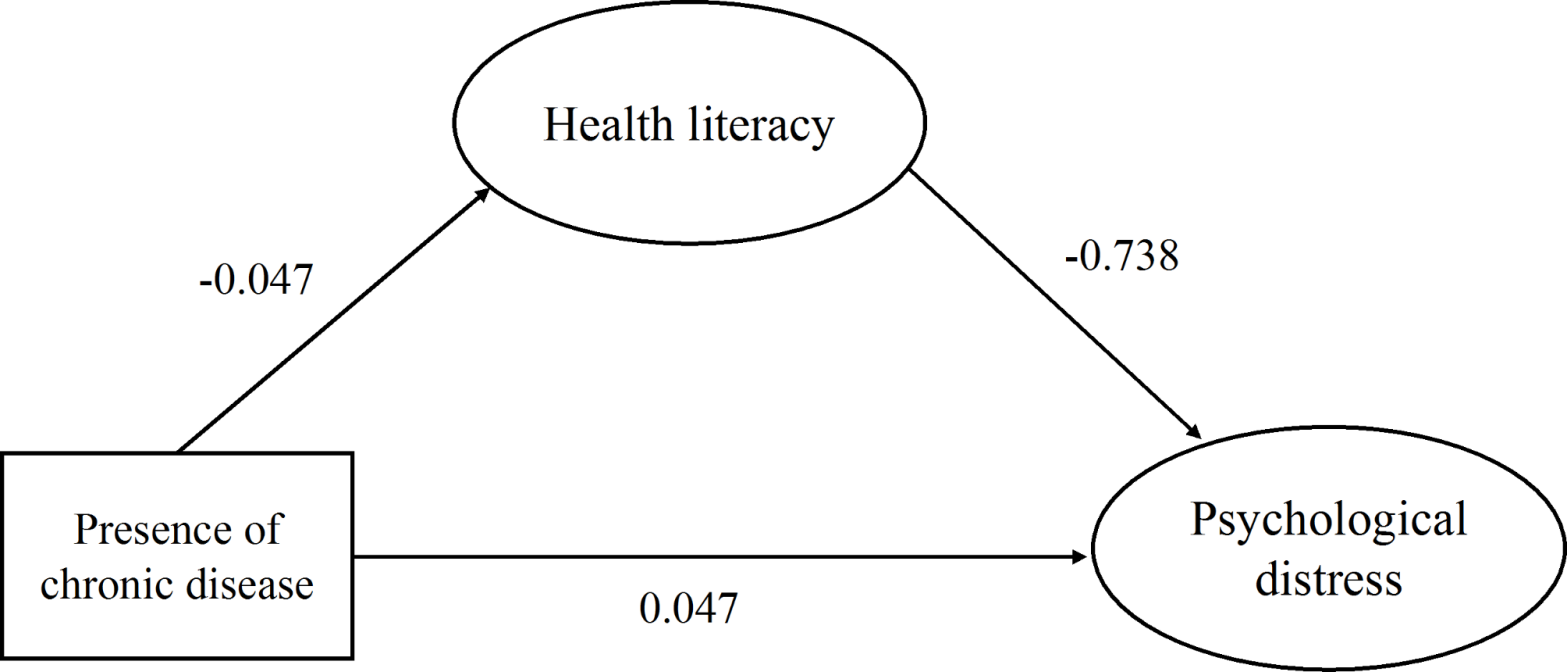
The final model and standardized model path coefficient

This study established a structural equation model, shown in Fig. 1, to examine how psychological distress would be influenced by the presence of chronic disease and health literacy. In the case of the final analysis, it has been established that the factor loading, which is determined in the structural model, was significantly higher than 0.6. The final output model is shown in Fig. 2, which presents the standardization path coefficient. The model fit indices of the hypothesized model all met the fitness criteria (CFI = 0.910, which was ≥ 0.90; TLI = 0.906 which was ≥ 0.90; RMSEA = 0.038, which was ≤ 0.08; SRMR = 0.063, which was ≤ 0.08 and Chi-sq/df = 1.43 which was ≤ 5.0).

The structural hypothesis is tested with bootstrapping corresponding to the method of 1000 replications [51]. Bootstrapping is a nonparametric approach that makes no distributional assumptions as to variable distributions and allows researchers to estimate standard errors and confidence intervals along with testing research hypotheses [53]. The standardized regression weights to answer the hypotheses are presented in Table 6. The presence of chronic disease has a direct impact on health literacy (β=-0.047, *p* < 0.05). The presence of chronic disease has a direct effect on psychological distress (β = 0.047, *p* < 0.05). Health literacy directly affects on psychological distress (β=-0.738, *p* < 0.001). Health literacy as the mediator effect on the association between the presence of chronic disease and psychological distress (β=-0.007, *p* < 0.001). With respect to the influence of the presence of chronic disease on psychological distress, we found the total effect, direct effect, and indirect effect of this path. These effects were all statistically significant, suggesting that the presence of chronic disease influenced psychological distress independently and partially through health literacy.

**Table 6:** Examining results of hypothesized correlation of the variables

Path	Path coefficient	Z value	*P* value	Hypothesis	95% Confidence intervals
H_1_: Presence of chronic disease → Health literacy	-0.047	-2.716	0.007	Accept	(-0.082, -0.002)
H_2_: Presence of chronic disease → Psychological distress	0.047	2.762	0.006	Accept	(0.009, 0.078)
H_3_: Health literacy → Psychological distress	-0.738	-5.667	< 0.001	Accept	(0.168, 0.489)
H_4_: Presence of chronic disease → Health literacy → Psychological distress	0.07	2.562	0.007	Accept	(0.045, 0.128)

## Discussion

This study determined health literacy as the mediator effect on the association between the presence of chronic disease and psychological distress among older persons in Xi’an city for the first time, thereby employing the theoretical support of CBT.

Of the 300 respondents in Xi’an city, this study found that 53.3% had a chronic disease, and 46.7% did not have a chronic disease. Patients with chronic diseases usually experience longer treatment, cost public health services much money, including pharmacological therapies, and are difficult to cure [54]. Chronic diseases account for about 70% of the burden in China, while chronic disease-related deaths account for 86.6% of total mortality. Chronic diseases were more prevalent among the participants in this study, in keeping with findings from a previous study that showed rapid urbanization and globalization of unhealthy lifestyles are contributing to the rise in chronic disease prevalence in China [55].

The Health Literacy Questionnaire (HLQ) was developed in Australia, and it was tested on diverse individuals in many countries’ communities to assess health literacy [24]. This study uses the Chinese version of HLQ to describe the health literacy profile, and it has strong validity and high reliability in assessing health literacy among older persons [26]. The nine-scale health literacy questionnaire can be administered to older persons to understand the multidimensional area of health literacy. The psychological distress subscales of depression, anxiety, and stress as indicators of the latent variable were effective. The current study provided substantial evidence regarding the convergent, composite reliability, and discriminant validity of HLQ and DASS-21 through CFA.

In this study, the presence of chronic disease was identified as the most significant factor that significantly influenced health literacy among older persons who live in Xi’an city (β=-0.047, *p* < 0.01). The findings of this study were consistent with the previous study, which proved Limited health literacy is negatively associated with the use of preventive diseases, management of chronic diseases, and self-reported health, and health literacy is associated with an increase in preventable hospital visits and admissions [56]. Another study found limited health literacy regarding instructions about misunderstanding prescription medication, medication errors, poor comprehension of nutrition labels, and mortality [57].

In this study, the presence of chronic disease was identified as the most significant factor that significantly influenced psychological distress among older persons who live in Xi’an city (β = 0.047, *p* < 0.01). This result was consistent with the previous studies, which have reported an association between chronic disease and psychological distress [54, 58], and the higher prevalence of chronic diseases’ negative effect on mental health and quality of life among older persons [59]. Therefore, it is critical to determine the relationship between the presence of chronic disease and psychological distress among older persons in Xi’an city.

This study identified health literacy as influencing psychological distress among older persons in Xi’an city (β=-0.738, *p* < 0.001). It indicated that an individual’s health literacy is an effective factor influencing psychological distress among older persons living in Xi’an city. The findings of this study, namely that health literacy direct effects the psychological distress of older persons in Xi’an, are consistent with the research findings that individuals with lower health literacy receive poorer quality of health care and poorer health outcomes [60]. Limited health literacy in older persons may adversely affect interpretations of health-related information and delay the use of mental health services and treatments.

This study found health literacy as the mediator between the presence of chronic disease and psychological distress (β = 0.07, *p* < 0.01). A mediator is an explanatory link in the relationship between two other variables. The mediator must be a causal result of the independent variables and a causal antecedent of the dependent variables. A previous study has found functional health literacy as a mediator in the pathway through which socioeconomic status affect health outcome [61]. A previous study found pain as a partially mediating effect on chronic disease and depression [62]. This study is the first attempt to determine the mediator effect on the association between the presence of chronic disease and psychological distress. It also suggested that improved health literacy should be considered when treating patients with psychological distress.

The strengths of this survey were that we focused on a rapidly growing vulnerable population – older persons and used trained interviewers in face-to-face interviews. The findings also offer the first empirical evidence of health literacy as the mediating role of the relationship between the presence of chronic disease and psychological distress. However, the results were interpreted cautiously due to several limitations. First, it was impossible to determine the causality of the variables (presence of chronic disease, health literacy, psychological distress) in this study because the research was cross-sectional. Second, the questions in the questionnaire, such as chronic diseases, were self-reported and not confirmed by a medical professional. Third, the instrument of DASS-21 for assessing depression, anxiety, and stress is only a screening tool for depression, anxiety, and stress rather than a diagnostic instrument. Therefore, we studied the association between the factors associated with mental health symptoms and mental illness. Fourth, the participants in our study come from six urban districts of Xi’an city in China, which might not reflect the actual situation related to the real condition of older persons in China. Fifth, in this study, health literacy cannot assess the level of health literacy. Lastly, data provided by Chinese participants may not be generalizable to other countries with different cultures.

The purpose of this study was to understand health literacy as the mediator role between the presence of chronic disease and psychological distress among older persons in Xi’an city. Several implications were drawn from the results of the present study that were specifically classified as theoretical and practical implications. The current research helps to address the lack of theoretical and empirical validation in this field. This study describes the mediating effect of health literacy between the presence of chronic disease and psychological distress among older persons in Xi’an City and evaluates the suitability of using a theory of CBT. Furthermore, the mediating effect of health literacy is a significant finding and contribution through the health literacy link between the presence of chronic disease and psychological distress according to CBT among older persons in Xi’an City. Thus, providing some measures to enhance health literacy might improve their ability to lighten their psychological distress. The findings from this study suggested health care providers should be on the lookout for signs of chronic disease with psychological distress in older persons, and public health management and the institutions that provide health care for older persons should provide more education about health literacy.

While this study was a cross-sectional study, longitudinal studies will in the long run help determine the causal relationship between the factors associated with psychological distress among older persons. Because this study was conducted through questionnaires based on older people’s self-report, there may be some bias resulting from the information found in their reports. In this study, the onset and severity of chronic illnesses were not assessed and only a list of chronic illnesses was assessed. It would be helpful to track the onset and severity of chronic illnesses in future studies to elucidate the findings. This study only focuses on the presence of chronic disease and dose not explore the different types of chronic disease and comorbidities. Future studies could add the comorbidities as a new variable to explore the severity of the presence of chronic disease. In the future, data collection may rely on medical assistance or an official medical mechanism. As mentioned earlier, all the respondents to this study were older than 60. Living conditions may also contribute to psychological distress among older persons, such as those who live alone or with their children [63]. Hence, it is suggested that future studies add the living environment to the study. The present study was also focused on a single city in China. Therefore, it is suggested that future studies could include older persons located throughout China. The scope of the study can be expanded to include students, the general population, or a specific occupational population in future studies.

## Conclusion

The effect of the presence of chronic diseases on the psychological distress of older persons can be partially predicted by determining their health literacy. This study emphasizes that psychological distress might be avoided in older persons with chronic diseases by improving health literacy. Therefore, health management that improves health literacy should be considered in the future treatment of older persons with both chronic diseases and psychological distress.

## Data Availability

The datasets generated during and analyzed during the current study are not publicly available due to the privacy but are available from the corresponding author on reasonable request.
